# The ePix10k 2-megapixel hard X-ray detector at LCLS

**DOI:** 10.1107/S1600577520004257

**Published:** 2020-04-17

**Authors:** Tim Brandt van Driel, Silke Nelson, Rebecca Armenta, Gabriel Blaj, Stephen Boo, Sébastien Boutet, Dionisio Doering, Angelo Dragone, Philip Hart, Gunther Haller, Christopher Kenney, Maciej Kwaitowski, Leo Manger, Mark McKelvey, Kaz Nakahara, Marco Oriunno, Takahiro Sato, Matt Weaver

**Affiliations:** a SLAC National Accelerator Laboratory, 2575 Sand Hill Road, Menlo Park, CA 94025, USA

**Keywords:** XFEL, detector, detector non-linearity, diffuse scattering

## Abstract

The ePix10ka2M (ePix10k) is a new large area detector specifically developed for X-ray free-electron laser (XFEL) applications. The hybrid pixel detector was developed at SLAC to provide a hard X-ray area detector with a high dynamic range, running at the 120 Hz repetition rate of the Linac Coherent Light Source (LCLS). The ePix10k consists of 16 modules, each with 352 × 384 pixels of 100 µm × 100 µm distributed on four ASICs, resulting in a 2.16 megapixel detector, with a 16.5 cm × 16.5 cm active area and ∼80% coverage. The high dynamic range is achieved with three distinct gain settings (low, medium, high) as well as two auto-ranging modes (high-to-low and medium-to-low). Here the three fixed gain modes are evaluated. The resulting dynamic range (from single photon counting to 10000 photons pixel^−1^ pulse^−1^ at 8 keV) makes it suitable for a large number of different XFEL experiments. The ePix10k replaces the large CSPAD in operation since 2011. The dimensions of the two detectors are similar, making the upgrade from CSPAD to ePix10k straightforward for most setups, with the ePix10k improving on experimental performance. The SLAC-developed ePix cameras all utilize a similar platform, are tailored to target different experimental conditions and are designed to provide an upgrade path for future high-repetition-rate XFELs. Here the first measurements on this new ePix10k detector are presented and the performance under typical XFEL conditions evaluated during an LCLS X-ray diffuse scattering experiment measuring the 9.5 keV X-ray photons scattered from a thin liquid jet.

## Abstract 2

The ePix10ka2M is a new large area detector specifically developed for X-ray free-electron laser applications. Here the detector parameters and performance are presented, as well as the detector nonlinearity as characterized by diffuse scattering measurements at the Linac Coherent Light Source.

## 1. Introduction   

X-ray free-electron lasers (XFELs) have opened the possibility to investigate ultrafast processes using extremely bright femtosecond X-ray pulses. The Linac Coherent Light Source [LCLS (Bostedt *et al.*, 2016 ▸)] was the first hard XFEL and has been in operation since 2009 (Emma *et al.*, 2010 ▸). The Cornell–SLAC Pixel Array Detector (CSPAD) (Philipp *et al.*, 2010 ▸, 2011 ▸; Herrmann *et al.*, 2013 ▸; Hart *et al.*, 2012 ▸; Carini *et al.*, 2014 ▸; Blaj *et al.*, 2015 ▸) large area detector was designed to meet the requirements of LCLS and has been in use since 2011. Most XFEL experiments require detectors that measure the X-ray intensities on a shot-to-shot basis. This defines the target requirements for a detector running at the repetition rate of the XFEL (120 Hz for LCLS) and the dynamic range to contain the maximum number of photons pixel^−1^ pulse^−1^ in a given experiment as well as allowing detection of experiment specific weak features. An example of such an experiment is the study of chemical dynamics through X-ray diffuse scattering (XDS) of molecules in solution, where a liquid jet is excited with an optical laser pulse and probed at different delays with diffuse hard X-ray scattering (van Driel *et al.*, 2016 ▸). Such XDS are typical experiments at the XPP (Chollet *et al.*, 2015 ▸) and XCS (Alonso-Mori *et al.*, 2015 ▸) endstations at LCLS which typically flood the detector with photons, but depend on averaging many shots to achieve sufficiently high signal-to-noise in order to extract sub-percent changes induced by the laser pulses. In order to extract such small changes in signal these experiments are extra sensitive to detector performance, linear­ity as well as artifacts that are not easily averaged out (van Driel *et al.*, 2015*a* ▸). This makes this type of experiment an excellent test of new detector performance.

## 2. Camera design   

**Figure 1:**
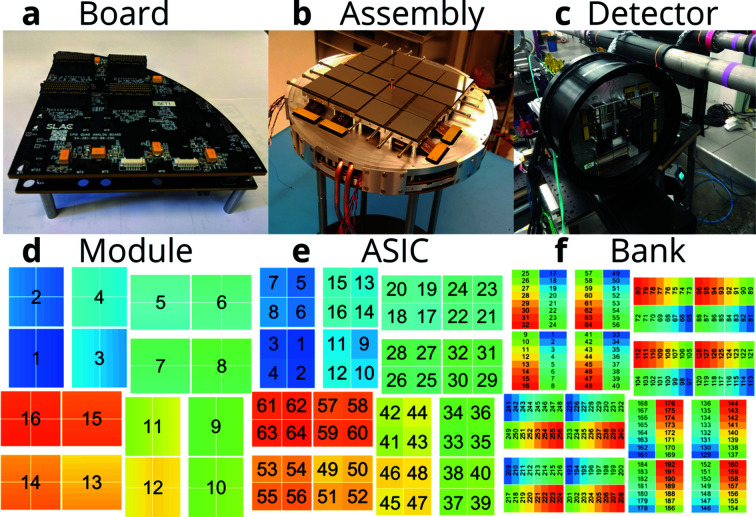
ePix10k detector layout. (*a*) Quadrant electronics boards. (*b*) Assembled modules, boards and cooling plates. (*c*) Full detector without a protective black Kapton screen. (*d*) Layout of the individual 16 modules. (*e*) Layout of the four ASICs on each module, 64 in total. (*f*) Layout of the 16 detector banks on each module, 256 in total.

The new SLAC-developed ePix10k detector [10 000 photons pixel^−1^ shot^−1^ saturation at 8 keV (Blaj *et al.*, 2019 ▸), 2 Mpixel detector], with high dynamic range has been commissioned and tested on a typical liquid experiment measuring the wide-angle X-ray diffuse scattering (WAXS, XDS) from a recirculating thin water jet probed with 9.5 keV XFEL pulses (van Driel *et al.*, 2016 ▸; Biasin *et al.*, 2016 ▸; Kong *et al.*, 2019 ▸; Kjaer *et al.*, 2019 ▸; Haldrup *et al.*, 2019 ▸). The new detector is evaluated in comparison with the CSPAD detector previously used, in order to evaluate the achievable performance in a typical XFEL experiment. The camera employs complex readout electronics partitioned into four quadrants (analog and digital board shown in Fig. 1*a* ▸). One quadrant reads out four ePix10k modules and 64 analog-to-digital converters (ADCs) are needed to achieve that (256 ADCs for the 16 modules in the full camera, one ADC per detector bank). The same multi-ADC chip was selected (AD9249 with 16 ADCs, 14 bits resolution, 65 MHz) that is used for the readout electronics of the small variant of the ePix10k camera [ePix detector type (Nishimura *et al.*, 2016 ▸)]. The large number of ADCs requires a field-programmable gate array (FPGA) with a large number of inputs/outputs (IOs); therefore a Kintex Ultrascale was selected with over 400 IOs (four FPGA chips in full camera). The data are being readout via four optical links with total throughput of 40 Gbps. The boards are stacked on a water-cooled cold-plate that is thermally strapped to another (upper) cold-plate designated to cool the ASIC modules [shown in Fig. 1(*b*) ▸].

The outer dimensions of the ePix10k are very similar to the CSPAD making it straightforward to replace in existing setups [Fig. 1(*c*) ▸]. As shown in Figs. 1(*b)*–1(*d*) ▸, the full detector consists of 16 modules (Blaj *et al.*, 2019 ▸) each containing four ASICs with four banks per ASIC. As seen in some of the data, it is relevant to keep the detector layout in mind since the performance can be localized to specific components. As an example, the CM and detector noise is related to the individual banks and the entire ASIC number 51 was exhibiting weird behavior during the experiment and should be masked out.

## 3. Data handling   

**Figure 2:**
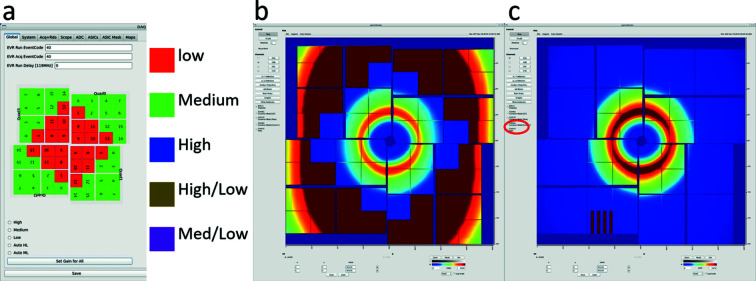
The AMI interface for the ePix10k detector. (*a*) The configuration can be used to easily configure individual ASICs in different gain modes and arbitrary gain masks can be uploaded with gain settings for the individual pixels; color code shown for assigned gain. (*b*) The detector configured in mixed gain mode as seen in (*a*). (*c*) The detector image after clicking correct gain, such that the image is correctly scaled to the gain so the actual intensity distribution can be visualized in real time.

The ePix10k is integrated into the LCLS data acquisition system (DAQ) and the gain mode of the individual ASICs can be configured as seen in Fig. 2(*a*) ▸ or an arbitrary pixel-wise gain map can be constructed and applied if something specific is desired. The GUI uses the color scheme in Fig. 2(*a*) ▸ for the different gain modes, and reconfiguration of the detector takes seconds. After changing the gain settings a new pedestal measurement should be recorded. For the data presented in this paper, the detector was configured in the fixed gain modes: low, medium and high gain as well as the fixed mixed gain presented in Figs. 2(*b*) and 2(*c*) ▸ where the ASICs exposed to the more intense scattering from the liquid ring is configured in low gain and the surrounding ASICs are configured in medium gain to achieve a better signal-to-noise for these pixels. At the time of the experiments, the auto-ranging gain modes were not fully implemented in online and offline data analysis and visualization, so only the fixed and mixed gain modes have been evaluated here. In the future, the auto-ranging medium-to-low or high-to-low gain modes will give better signal-to-noise for all pixels, even for a fluctuating signal as the gain simply switches from high to low gain as the individual pixel is exposed to a number of photons exceeding the threshold for the gain mode. The LCLS Analysis Monitoring Interface (AMI) (Thayer *et al.*, 2016 ▸) is used to monitor the detector output with the correct geometry in real time, as seen in Fig. 2(*b*) ▸, and the gain correction is applied by selecting the option on the GUI as seen in Fig. 2(*c*) ▸. This real-time view of the detector can also be used for fast online analysis of the data and is routinely used for alignment and optimization in a given experiment. The detector is implemented in the PSANA Python analysis framework (Damiani *et al.*, 2016 ▸) where the full data-sets can be analyzed shortly after the data are taken. This interface allows for deployment of detector corrections in Python scripts that can be applied or modified by the users, during or after an experiment.

Table 1 ▸ contains a comparison of many of the relevant parameters and performance numbers of the CSPAD and ePix10k detectors. The general dimensions and parameters are rather similar for the two detectors. The ePix10k is a 2.16 megapixel detector constructed of 16 modules arranged to form a larger 2D area detector with gaps between modules resulting in 80% coverage of a 16.5 cm × 16.5 cm area. The CSPAD was designed to target a large variety of XFEL experiments and has two fixed gain modes [low (2000 photons), high (320 photons) at 9.5 keV] where the ePix10k detector has three fixed gain modes [low (8200 photons), medium (270 photons) and high (80 photons) at 9.5 keV] as well as medium-to-low and high-to-low auto-ranging which will not be presented here. The signal-to-noise performance of the ePix10k detector gives it a dynamic range from single photon counting (above 3.2 keV, since 5σ = 1.6 keV in high gain) up to 9300 photons pixel^−1^ frame^−1^ at 9.5 keV. This allows for the use of the full LCLS beam (1 × 10^12^ photons pulse^−1 ^ on sample resulting in 2 × 10^8^ photons frame^−1^ on the detector) for a typical diffuse scattering experiment on a 50 µm water sample with the ePix10k placed 6 cm downstream from the interaction point. The high dynamic range hereby allows simultaneous detection of bright pixels flooded by 1000s photons pixel^−1^ pulse^−1^ near the liquid ring as well as weakly illuminated pixels near the edges of the detector exposed to 1s photons pixel^−1^ pulse^−1^ with high fidelity.

### 3.1. Pedestal   

**Figure 3:**
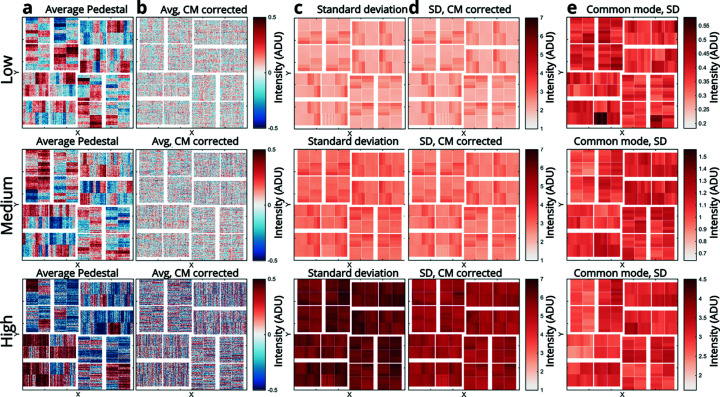
Pedestal performance for the three different gain modes: low gain (top), medium gain (middle) and high gain (bottom). (*a*) Average pedestal taken 15 min after a pedestal was recorded and applied. (*b*) Similar pedestal after CM correction. (*c*) Standard deviation of the recorded pedestal shots. (*d*) Standard deviation after CM subtraction. (*e*) Absolute mean of the CM in the individual banks mapped onto the detector.

The pedestal (also referred to as dark) was measured by recording a number of shots (typically 1000) while not exposing the detector. In addition a pedestal scrip exists that records the pedestal for all three gain modes for use with auto-ranging. The pedestal was determined by averaging such unexposed shots and the standard deviation is also calculated to be used to mask out statistically noisy pixels. The pedestal is temperature dependent, so giving the detector time to cool down (typically 15 min) and monitoring the temperature and cooling can be beneficial. When running the detector at 15°C, it stabilizes in minutes once the temperature is reached and the pedestal shows good stability over time. Fig. 3 ▸ shows the pedestal performance of the three fixed gain modes of the ePix10k detector. The pedestal and standard deviation of the raw data [Figs. 3(*a*) and 3(*c*) ▸] shows some local behaviour in the different banks [see Fig. 1(*d*) ▸]. In order to correct for the common mode (CM) (Blaj *et al.*, 2015 ▸; Pietrini & Nettelblad, 2017 ▸) behavior the average of each bank is subtracted in each shot, to extract the resulting noise as seen in the CM corrected average and standard deviation [Figs. 3(*b*) and 3(*d*) ▸]. From the CM corrected data it can be seen that the dominating source of noise [standard deviation, Fig. 3(*d*) ▸] is not the CM and that the noise has an intrinsic pattern related to the orientation of the individual modules. As given in Table 1 ▸, the noise performance in the different gain-modes was calculated as the mean of the noise distribution, given by the standard deviation of each pixel, calculated both before and after CM correction. While the CM fluctuations can be easily corrected when the number of incoming photons is low, the cases where the detector is flooded by photons can make this correction difficult. The magnitude of the CM fluctuations was determined [as given in Table 1 ▸ as well as Fig. 3(*e*) ▸] and, based on the numbers (181 eV, 221 eV and 2580 eV r.m.s. in high, medium and low gain, respectively), the CM can reasonably be ignored in the case where the detector sees many photons.

As with the CSPAD, the pedestal should be recorded whenever the detector is turned on, the gain mode is reconfigured or the temperature changes, so typically pedestals are recorded at least at the beginning and end of each shift (12 h) but typically pedestals are recorded a few more times during the shift, since it can be done without X-rays, and provides additional points of reference in case the pedestal changes over time. In addition to dedicated pedestal runs, the X-ray pulses are dropped periodically, and these shots can be used as internal pedestals throughout experiments to monitor the pedestal stability and as described below can help identify non-ideal detector behaviour.

### 3.2. Ghost images   

**Figure 4:**
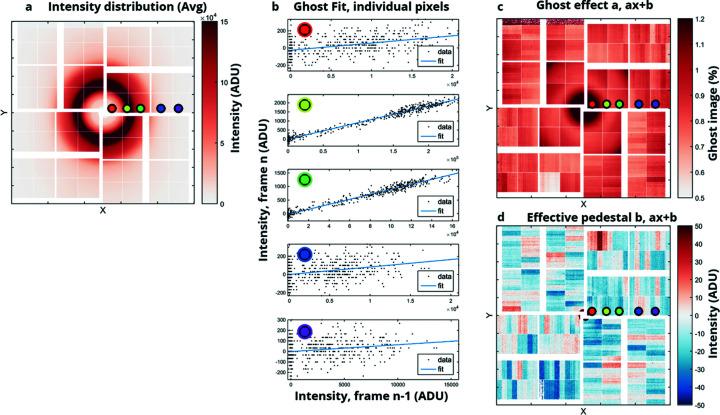
Ghost effect as observed on the ePix10k detector. (*a*) Example intensity distribution. (*b*) Linear fit of a dark frame and the frame before for the five chosen pixels at different relative intensities and locations (colored dots). (*c*) Resulting slope describing the magnitude of the ghost effect. (*d*) Small offset describing the effective pedestal.

Upon inspecting the unexposed frames recorded when periodically dropping the X-ray shots, a remnant of the previously exposed frame was observed. This effect was observed during an actual experiment where the ePix10k detector was exposed to the diffuse liquid scattering from a water sample. In the case of diffuse scattering from water, the intensity distribution remains constant for a stable setup and the presence of a ‘liquid ring’ in the dropped shots indicates that a ghost image’ is present. Upon further investigation this ghost image’ contains approximately 0.9% of the previous shot. The residual image originates in the readout circuit as it is incompletely cleared after each readout. For future ePix detectors this will be remedied in the ASIC design. The current ePix10k with the ghost image problem is designed to run at 360 Hz. A firmware fix has been implemented that reads out the detector at double the repetition rate (240 Hz) and therefore reduces the ghost image to 0.9% times 0.9% (*i.e.* 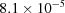) of the preceding signal, which is below the noise of the detector. This firmware fix has been implemented but not yet tested with XFEL beam on the detector. Meanwhile we have developed a software solution to account for the effect in recorded data. In order to account for the ‘ghost’ effect and subtract it in the recorded data, the ghost effect was quantified based on the relationship between the intensity observed in a pixel in an unexposed frame and the preceding exposed frame. As seen in Fig. 4 ▸, the resulting ghost effect for the intensity distribution shown in Fig. 4(*a*) ▸ can be determined by fitting the intensity relation for each pixel [see Fig. 4(*b*) ▸ for five example pixel fits]. To fit the ghost effect on a given pixel for a given intensity distribution, the intensity of the supposedly dark dropped shots is plotted against the intensity in the previous shot and fit as a linear dependency [equation (1)]. Fitting the ghost effect with a simple linear least-squares fit yielded good and fast results and did not differ greatly from the more robust Theil-Sen estimator (Gilbert, 1987 ▸) or a second-order polynomial fit, 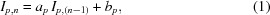
where 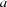 is the ghost effect and 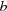 is the effective pedestal of pixel 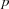. The magnitude of the ghost effect is 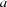 ≃ 0.9% but depends on the gain mode and intensity distribution. As seen from Figs. 4(a) and 4(*c*) ▸, the ghost effect seems to depend on the intensity gradient in a given ASIC, and therefore the resulting correction determined here can only be applied if the intensity distribution remains the same. In order to correct the effect, we run the ghost effect linear fitting on each recorded run, typically comprising 10 min of data (600 dropped shots for fitting) since the intensity distribution typically remains constant while recording data in a liquid diffuse scattering experiment. This post-processing removal of ghost images is necessary to remove the effect from data measured where the ghost image problem was present, but should not be necessary for future runs where either the firmware or hardware fix will be employed.

### 3.3. Linearity   

After subtracting the pedestal and correcting for the ghost images the linearity of the signal can be evaluated. Here we evaluate the linearity of the pixels on the ePix10k detector during an actual experiment. A 50 µm liquid water jet was recirculated and the scattering from this sample was repeatedly measured. By varying the incoming X-ray intensity using solid attenuators and keeping the sample constant it is possible to measure the detector response with a constant intensity distribution covering the dynamic range of the detector. Assuming that the sample is stable and repeatable any fluctuations in the signal shape are due to the X-ray beam or the detector. As observed for a similar setup on the CSPAD (van Driel *et al.*, 2015*a* ▸) the dominating effect was the non-linear intensity dependency of the detector. For a stable and repeatable sample and X-ray beam that only changes in intensity, the intensity distribution should be constant and the signal simply scale with the number of incoming X-ray photons and any observed deviations from linearity due to the detector. The intensity from a SASE source fluctuates significantly on a shot-to-shot level, especially after monochromatization (Zhu *et al.*, 2014 ▸). As described in previous work, the effect of the beam fluctuations and resulting effect on the detector images can be corrected as long as the non-linearities are reproducible and X-ray beam parameters are recorded (van Driel *et al.*, 2015*a* ▸,*b* ▸).

**Figure 5:**
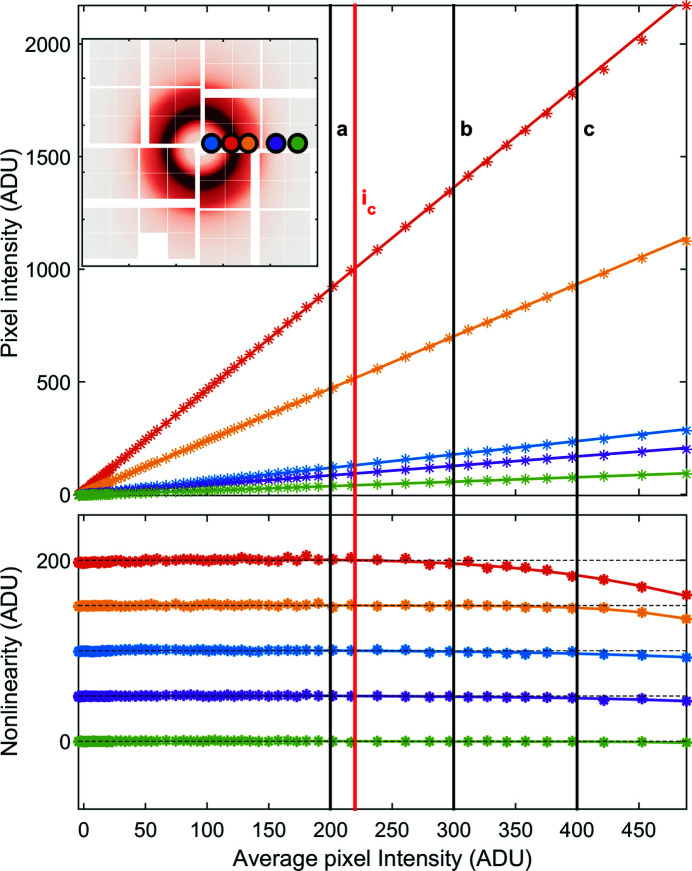
Linearity of the ePix10k detector in low gain. Top panel: the intensity of five chosen pixels when varying the incoming X-ray intensity to show the deviation from a linear response at different intensities across the detector (indicated by the colored dots in the insert showing the intensity distribution of the scattering from water). The data from multiple acquired images were binned based on the average intensity on the detector. The solid black lines *a*,*b*,*c* represent selected intensities where the correction is evaluated in Fig. 6 ▸. Bottom panel: nonlinear residuals after subtracting the first-order polynomial fit around a chosen correction intensity 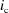. The residuals from each pixel have been offset for visibility.

The ghost corrected ePix10k data were binned according to average intensity on each image. As shown in Fig. 5 ▸, the binned intensity of each pixel was fit with a first-order polynomial (Fig. 5 ▸, top) to find the deviation from linearity (Fig. 5 ▸, bottom). A detailed description as well as example code has been given by van Driel *et al.* (2015*b* ▸). This approach allows us to fit a polynomial to the individual pixel behaviour and use the derivative to correct the measured intensity to a linear detector response determined around a chosen intensity 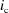. This correction is intensity distribution dependent and therefore requires the recorded images to have an identical or similar intensity distribution to be valid and effective. In the case of liquid diffuse scattering this is almost always the case as the sample is replenished continuously and the desired laser induced difference signal is small (∼1%) and therefore does not drastically change the intensity distribution.

The average intensity on the detector is used as 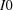 describing the incoming intensity. This is not ideal since given a non-linear detector response 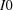 will also be non-linear and will underestimate the magnitude of the non-linearity. However, though the absolute magnitude of the non-linearity may be underestimated, the shape of the non-linearity is not effected and the ability to correct the effect is not effected either. In addition the average intensity of the detector has much better statistics and measures the incoming intensity on the detector given by the sample and incoming beam, where any upstream diagnostics do not account for sample fluctuations.

**Figure 6:**
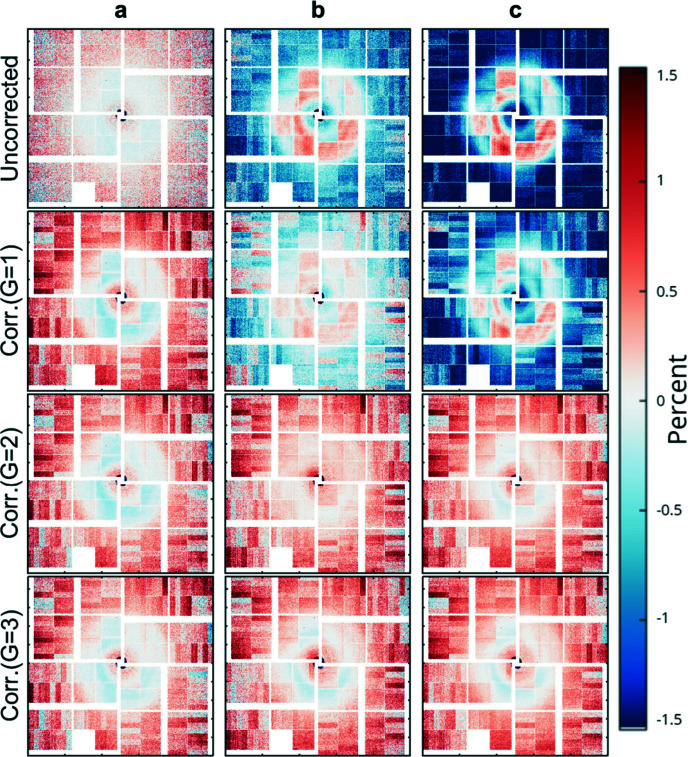
Effect of the non-linear intensity behaviour of the ePix10k detector in low gain before and after applying the corrections described here and in more detail by van Driel *et al.* (2015*b* ▸) on an ePix10k dataset containing diffuse liquid scattering from a water sample as seen in th einsert of Fig. 5 ▸. The images at three different intensities were evaluated in relation to the image around reference intensity 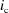. The resulting deviation 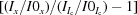 is shown for the three reference intensities 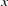 = *a*,*b*,*c* for the uncorrected data as well as data corrected with a polynomial of order *G* = 1,2,3 showing the need for at least a second-order polynomial to successfully correct most of the non-linear detector dependency of the ePix10k.

Comparing the normalized image, 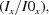 at a given intensity 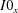 with the normalized reference image 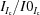 allows us to evaluate the magnitude of the non-linear effect. If the detector exhibited ideal behavior the ratio given by 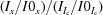 ≃ 1 and would simply contain a contribution from the Poisson noise of the averaged images. This ratio 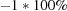 allows us to evaluate the non-linear effect as a percentage as well as the effect of applying corrections using a polynomial of order 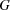 at three chosen different intensities *a*, *b*, *c* (as shown in Fig. 6 ▸). As can be seen, the non-linear effect introduces a ∼1.5% error at higher intensities and can be effectively corrected with a *g* = 2 second-order polynomial description of the non-linear intensity dependent behaviour.

## 4. Conclusion   

The ePix10k detector has been tested at LCLS under experimental conditions. The three fixed gain modes (high, medium and low) were evaluated and all showed good performance in agreement with the previously tested single module (Blaj *et al.*, 2019 ▸). The pedestal was observed to show good stability over time and the noise was observed to differ slightly across different banks. Upon investigating unexposed frames following bright frames a ghost image effect was observed on the ∼0.9% level. This ghost image dependency was further observed to depend on the intensity distribution on the detector. Fitting the ghost image as a pixel-by-pixel intensity-dependent effect based on the regularly dropped X-ray shots and the previous exposed frame allows for post-correction of this effect. In the future the ghost effect will be removed by a firmware update that will run the detector at twice the repetition rate (240 Hz instead of 120 Hz, effectively clearing the detector with a disposable frame) or by a future hardware upgrade updating the ASICs such that the readout circuit is cleared between consecutive frames. After removing the pedestal and correcting for the ghost image effect, the linearity of the detector was evaluated in the large dynamic range that can only be tested at an XFEL due to the need for extremely high flux allowing us to flood the detector with photons. The nonlinear intensity dependency was determined to be on the order of 1.5% and can be effectively corrected with a second-order correction (van Driel *et al.*, 2015*b* ▸). The ePix10k detector shows good performance in the fixed gain modes, and future efforts will focus on carefully characterizing the auto-ranging performance. Pixelwise auto-ranging on a shot-to-shot basis was recently developed for X-ray and other ionizing radiation detection (Freytag *et al.*, 2008 ▸; Brau *et al.*, 2012 ▸), and shows great promise to extend the dynamic range and improve the signal-to-noise for XFEL purposes (Allahgholi *et al.*, 2015 ▸; Mozzanica *et al.*, 2016 ▸).

Based on the charcaterization presented here, the ePix10k is well suited to replace the large CSPAD in a multitude of applications to supply better noise performance and larger dynamic range. The ePix detector architecture also provides a pathway to producing such detectors for future higher=repetition=rate facilities such as LCLS-II and LCLS-II-HE (Blaj *et al.*, 2016 ▸).

## Figures and Tables

**Table 1:** CSPAD *versus* ePix10k parameters and performance numbers in the different fixed gain modes presented in this paper. The autoranging modes of the ePix10k are not presented here

Detector	CSPAD		ePix10k		
Megapixels	2.3		2.16		
Pixel size (µm)	110 × 110		100 × 100		
Size (cm)	18.6 × 18.6		16.5 × 16.5		
Coverage	80%		80%		
Bit depth	14 bit		14 bit		
Gain mode	High	Low	High	Medium	Low
Gain factor	1	1/6	1	1/3	1/100
Pedestal (ADU)	1500		3100		
Gain (ADU/9.5 keV photon)	46	7.6	162	48.6	1.62
Saturation (9.5 keV photon)	320	2000	80	270	8200

eV/ADU	206.5	1250	58.5	195	5864

Raw r.m.s. (ADU) Fig. 3(*c*)	4.78	2.18	6.32	3.09	2.36
Noise r.m.s. (ADU) Fig. 3(*d*)	4.34	2.10	5.54	2.88	2.33
Common mode r.m.s. (ADU) Fig. 3(*e*)	2.03	0.58	3.08	1.13	0.44

Raw r.m.s. (eV)	987	2725	371	603	13840
Noise r.m.s. (eV)	896	2625	325	562	13664
Common mode r.m.s. (eV)	419	725	181	221	2580

Signal-to-noise (9.5 keV)	10.6	3.6	29.2	16.9	0.7
Signal-to-noise (9.5 keV) without CM removal	9.6	3.5	25.6	15.7	0.7
